# RBAP, a Rhodamine B-Based Derivative: Synthesis, Crystal Structure Analysis, Molecular Simulation, and Its Application as a Selective Fluorescent Chemical Sensor for Sn^2+^

**DOI:** 10.3390/molecules19067817

**Published:** 2014-06-11

**Authors:** Xiaofeng Bao, Xiaowei Cao, Xuemei Nie, Yanyan Jin, Baojing Zhou

**Affiliations:** 1 Department of Biochemical Engineering, Nanjing University of Science & Technology, Chemical Engineering Building B308, 200 Xiaolinwei, Nanjing 210094, China; E-Mails: caoxiaowei518@126.com (X.C.); j.yy.1002c@163.com (Y.J.); 2 Department of Chemistry, School of Chemical Engineering, Nanjing University of Science & Technology, Nanjing 210094, China; E-Mails: xuemeinie@163.com (X.N.); bzhou@njust.edu.cn (B.Z.)

**Keywords:** rhodamine B, RBAP, Sn(II) ion, chemical sensor

## Abstract

A new fluorescent chemosensor based on a Rhodamine B and a benzyl 3-aminopropanoate conjugate (**RBAP**) was designed, synthesized, and structurally characterized. Its single crystal structure was obtained and analyzed by X-ray analysis. In a MeOH/H_2_O (2:3, v/v, pH 5.95) solution **RBAP** exhibits a high selectivity and excellent sensitivity for Sn^2+^ ions in the presence of many other metal cations. The binding analysis using the Job’s plot suggested the RBAP formed a 1:1 complex with Sn^2+^.

## 1. Introduction

**Figure 1:**
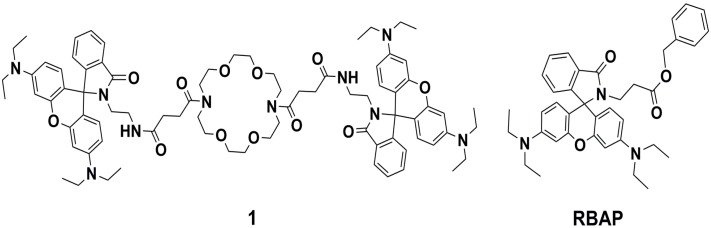
Chemical structures of **1** and **RBAP**.

Selective and sensitive fluorescent sensors for the detection and quantification of transition-metal ions are widely attractive to current researchers because of their simplicity, high sensitivity and instantaneous response [1,2,3,4,5,6,7]. Conventional methods used to detect metal ions usually require large and expensive instruments and include atomic absorption/emission spectrometry [8,9,10], ion-coupled plasma emission-mass spectrometry [11] and X-ray fluorescence spectroscopy [12,13]. These instrumentally intensive methods often also require extensive sample preparation prior to analysis and sophisticated experimental procedures [14]. Thus, a simple and inexpensive method for detecting and quantifying metal ions is essential for real-time monitoring in biological samples. Ions of tin (Sn), a type of heavy metal, are usually found in the environment at low levels. Humans are usually exposed to organic tin complexes through packaged foods, soft drinks, biocides, and dentifrices [15]. Little attention has been paid to the toxicity of Sn^2+^ as an environmental pollutant in natural waters. Although tin is not a highly toxic element [16], at high concentrations of approximately 0.1–1.0 g/L, Sn^2+^ may affect water flavor and cause diarrhea. Recent literature reports have revealed that, in forms such as SnCl_2_, Sn^2+^ can be readily taken up by human white blood cells and cause DNA damage [17,18]. Thus, it is desirable to develop a reliable and sensitive analytical method to qualitatively and quantitatively evaluate the level of Sn(II) ions present in environmental and biological systems. Rhodamine B derivatives are extensively employed as molecular probes in the study of complex biological systems due to their high absorption coefficients, high fluorescence quantum yields, and long-wavelength absorptions and emissions [19,20]. On the basis of the spirolactam/ring-opened amide equilibrium of rhodamine, several fluorescence-based sensing systems for metal ions have been developed. Most of the reported sensors based on rhodamine B derivatives are fluorescent chemosensors for the detection of Pb^2+^, Cd^2+^, Cu^2+^, Fe^3+^ and Hg^2+^ ions [21,22,23,24,25,26,27,28,29]. Unfortunately, to the best of our knowledge, only a few rhodamine B-based chemosensors have been reported for tin ions [30]. Therefore, a rhodamine B architecture was selected for the development of a new chemosensor that can selectively detect Sn^2+^. Recently, we reported rhodamine B-based sensor **1** (Figure 1), which consists of two rhodamine B moieties linked through the two amines of a 4,13-diaza-18-crown-6 ether as a highly sensitive fluorescent probe for monitoring Cr^3+^ in a MeOH/H_2_O (3:2, v/v, pH 7.2) solution and in living cells [31]. Unfortunately, single crystals of sensor **1** could not be grown in the presence or absence of Cr^3+^ and were not analyzed by X-ray diffraction. In this paper, we report the design and synthesis of a rhodamine B–based derivative bearing a benzyl 3-aminopropanoate group (**RBAP**) that selectively displays a colorimetric response and a fluorescence “turn-on” response at 583 nm via a rhodamine ring-opening process with Sn^2+^ in the presence of many other metal ions. We also report the single crystal X-ray analysis of **RBAP**, which further confirms its structure in the absence of Sn^2+^. Additionally, a DFT computational study was carried out for better understanding of the formation of a complex between **RBAP** and Sn^2+^. To the best of our knowledge, **RBAP** is the first rhodamine B-based sensor for the Sn^2+^ ion utilizing the rhodamine ring-opening equilibrium approach.

## 2. Results and Discussion

### 2.1. Synthesis of **RBAP**

**Scheme 1:**
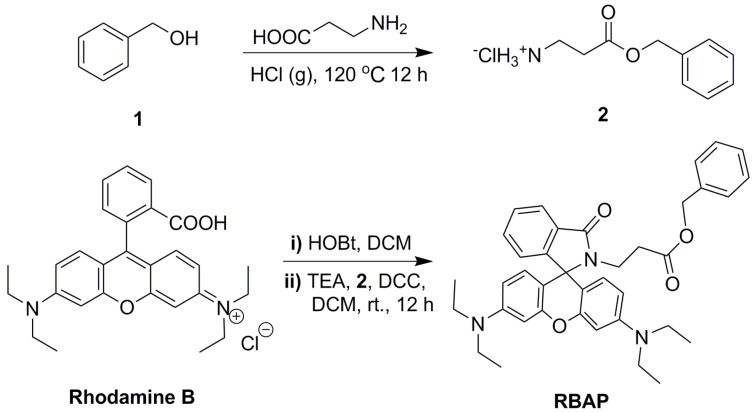
Synthesis of **RBAP**.

**RBAP** was synthesized according to the procedure published in the literature [31] (Scheme 1). Specifically, compound **2** was prepared in a 92% yield by treating **1** with 3-aminopropanoic acid and HCl gas at 120 °C for 12 h, which was followed by coupling with rhodamine B in the presence of DCC (1 eq.), HOBt (1 eq.), and TEA (3 eq.) in CH_2_Cl_2_ at room temperature for 12 h to give **RBAP** in a 90% yield. 

### 2.2. X-ray Crystallographic Analysis

**Figure 2:**
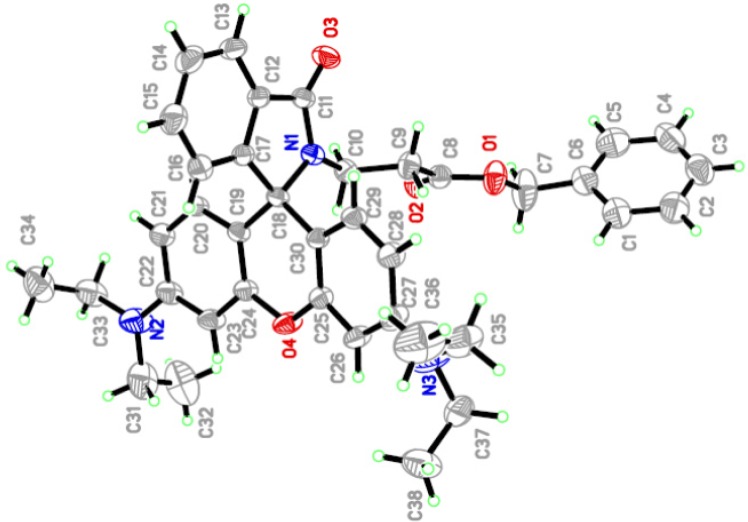
Crystal structure of **RBAP**.

**Figure 3:**
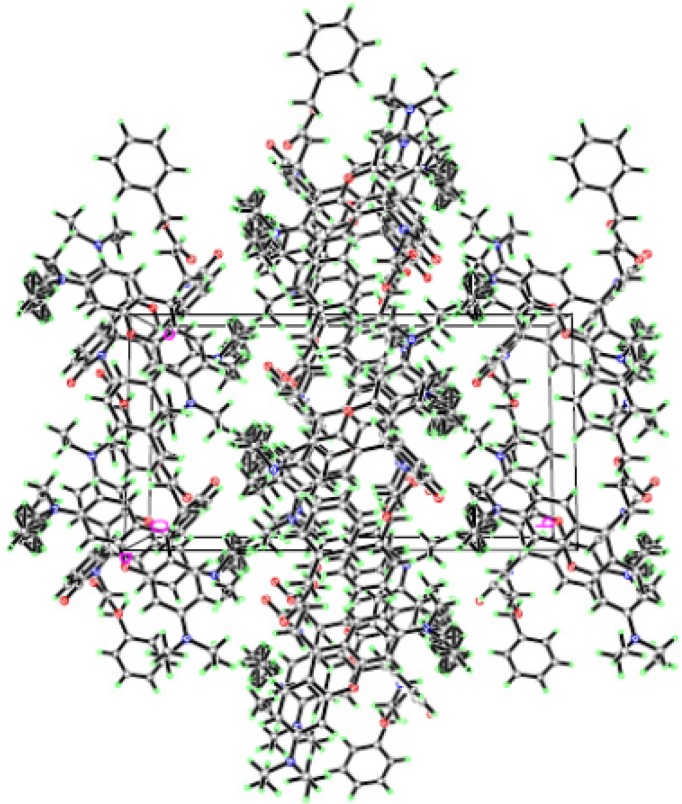
Network structure of **RBAP**.

A single crystal of **RBAP** suitable for X-ray diffraction studies was grown by vapor diffusion of a CH_2_Cl_2_ solution of **RBAP**. The chemical structure of **RBAP** was further confirmed by X-ray analysis as shown in Figure 2, and the two-dimensional network structure of **RBAP** is shown in Figure 3.

### 2.3. pH Response of **RBAP**

**Figure 4:**
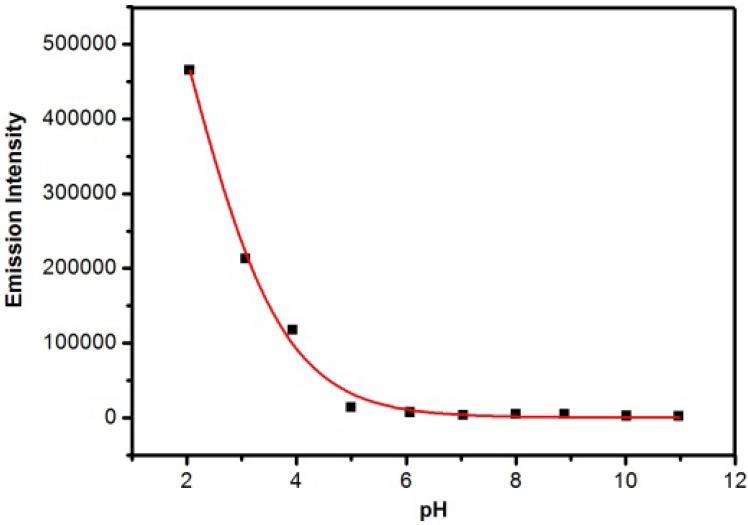
The influence of pH on the fluorescence of **RBAP** in MeOH/H_2_O solutions (2:3, v/v), pH was modified by adding 10% HCl or 10% NaOH.

**Scheme 2:**
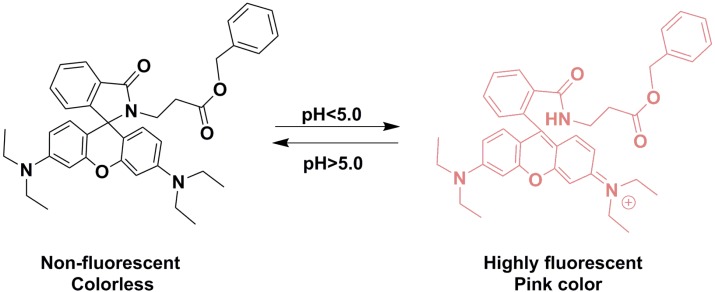
Mechanism of **RBAP** response to pH.

To evaluate the pH response of **RBAP**, acid-base titration experiments were performed in a MeOH/H_2_O (2:3, v/v, pH 5.95) solution. The fluorescence intensities of **RBAP** at 583 nm in solutions at different pH levels were recorded. As shown in Figure 4, **RBAP** did not emit any obvious and characteristic fluorescence (excitation at 561 nm) in the pH range of 5.0 to 12.0. However, the fluorescence intensity at 583 nm was obviously enhanced at pH levels below 5.0 due to the ring-opening mechanism of the spirocyclic moiety of rhodamine B (Scheme 2). These results suggested that **RBAP** was insensitive to pH from 5.0 to 12.0 and may have been able to sense Sn^2+^ under approximate physiological conditions with very low background fluorescence. Therefore, further UV-Vis and fluorescence studies were carried out in a MeOH/H_2_O (2:3, v/v, pH 5.95) solution.

### 2.4. UV-Vis Titration of **RBAP** with Metal Ions

**Figure 5:**
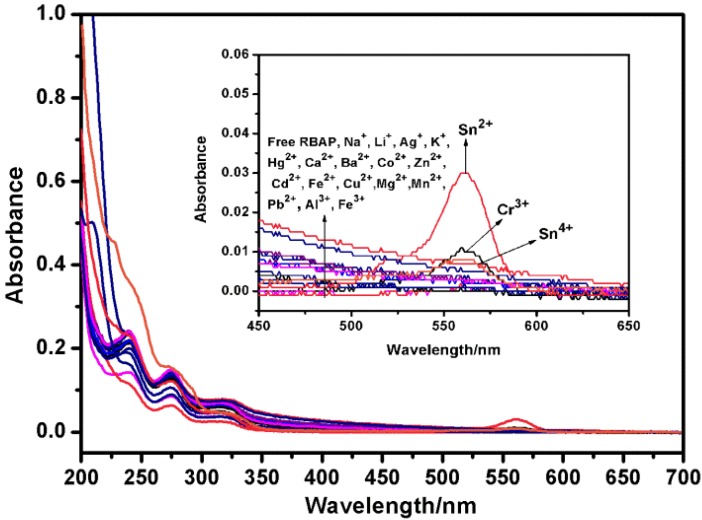
UV-Vis spectra of RBAP (10 μM) in MeOH/H_2_O (2:3, v/v, pH 5.95) in the presence and absence of metal cations (50 μM, 5.0 equiv.): Mg^2+^, Sn^2+^, Cr^3+^, Ag^+^, Ca^2+^, Na^+^, Pb^2+^, K^+^, Mn^2+^, Zn^2+^, Cu^2+^, Cd^2+^, Li^+^, Ba^2+^, Fe^2+^, Co^2+^, Fe^3+^, Hg^2+^, Al^3+^ and Sn^4+^.

To evaluate the selectivity of **RBAP** for the Sn^2+^ ion, the binding behavior of **RBAP** toward different metal cations (Mg^2+^, Sn^2+^, Cr^3+^, Ag^+^, Ca^2+^, Na^+^, Pb^2+^, K^+^, Mn^2+^, Zn^2+^, Cu^2+^, Cd^2+^, Li^+^, Ba^2+^, Fe^2+^, Co^2+^, Fe^3+^, Hg^2+^, Al^3+^, Sn^4+^) was studied by UV-Vis spectroscopy. All measurements were made according to the following procedure. Test samples were prepared by placing five equivalents of a metal ion stock solution into 10 μM **RBAP** in MeOH/H_2_O (2:3, v/v, pH 5.95) (3 mL) and UV absorption spectra were measured 30 min after metal ion addition. All titration experiments were recorded at room temperature. The absorption wavelength for **RBAP** was 561 nm. Of the various metal cations examined, **RBAP** showed a highly selective “off-on” absorption enhancement with Sn^2+^ at 561 nm. Cr^3+^ also resulted in a small absorbance at 561 nm, but at a 3-fold lower intensity than Sn^2+^. All other metal cations yielded very little absorbance at 561 nm (Figure 5).

**Figure 6:**
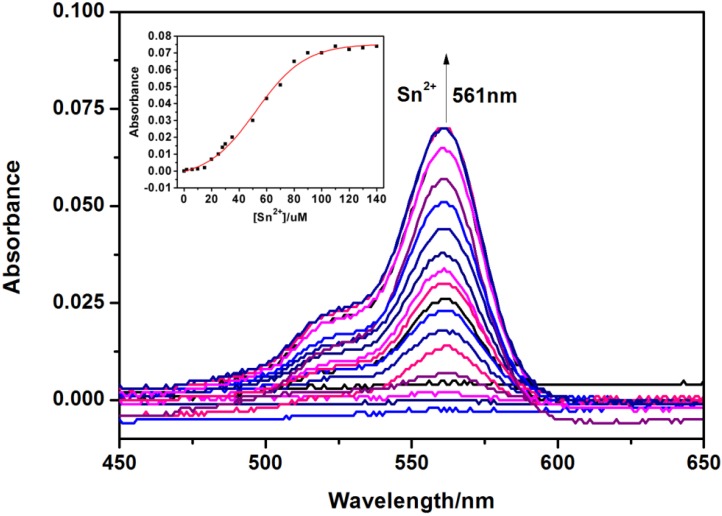
Changes in the UV-Vis absorption spectra of RBAP (10 μM) in MeOH/H_2_O (2:3, v/v, pH 5.95) solutions containing various amounts of Sn^2+^ ions (0–15 eq.). Inset: Absorbance of RBAP at 561 nm as a function of Sn^2+^ concentration.

The absorption spectra of **RBAP** upon titration with Sn^2+^ in MeOH/H_2_O (2:3, v/v, pH 5.95) solution were recorded to gain further insight into the binding of **RBAP** and Sn^2+^. When no Sn^2+^ ions were added to the solution of **RBAP**, the free **RBAP** remained colorless and did not exhibit any apparent absorption above 400 nm in MeOH/H_2_O (2:3, v/v, pH 5.95) solution, indicating that the spirolactam form of **RBAP** was the predominant species. Upon addition of the Sn^2+^ ion, a new and strong absorption band centered at 561 nm was observed (Figure 6), resulting in the color change from colorless to pink. This indicated that the ring-opened form of **RBAP** exists in a significant concentration in the examined solution (Scheme 2).

**Figure 7:**
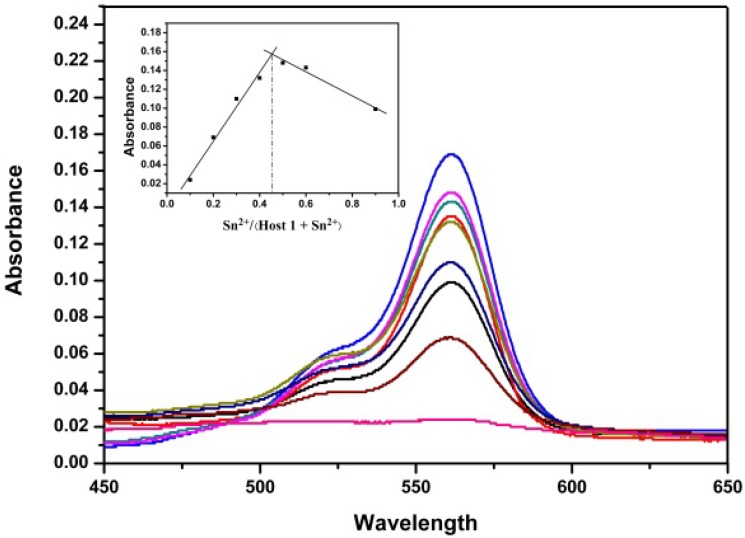
Job’s plot of **RBAP** in a MeOH/H_2_O (2:3, v/v) solution, with a total concentration of [**RBAP**] + Sn^2+^ = 100 μM and a detection wavelength of 561 nm.

Next, a Job’s plot was generated by continuously varying the mole fraction of Sn^2+^ from 0 to 1 in a solution of [Sn^2+^] + [**RBAP**] with a total concentration of 100 μM. The Job’s plot analysis exhibited a maximum at approximately 0.5 mole fraction, indicating a 1:1 stoichiometry for the **RBAP**-Sn^2+^ complex (Figure 7 inset).

### 2.5. Fluorescence Titration of **RBAP** with Metal Ions

**Figure 8:**
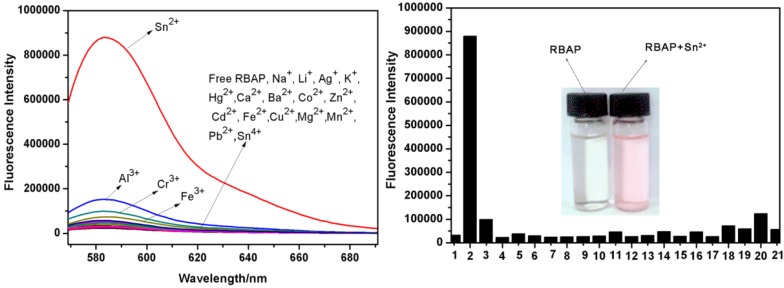
Fluorescence emission changes of RBAP (10 μM) with metal cations (50 μM, 5.0 equiv.) in MeOH/H_2_O (2:3, v/v, pH 5.95) at 583 nm, Black bars represent the fluorescence intensity of RBAP with: 1-none; 2-Sn^2+^; 3-Cr^3+^; 4-Ca^2+^; 5-Ag^+^; 6-Mg^2+^; 7-K^+^; 8-Ba^2+^; 9-Co^2+^; 10-Zn^2+^; 11-Li^+^; 12-Cd^2+^; 13-Fe^2+^; 14-Cu^2+^; 15-Mn^2+^; 16-Pb^2+^; 17-Na^+^, 18-Fe^3+^, 19-Hg^2+^, 20-Al^3+^, 21-Sn^4+^.

**Scheme 3:**
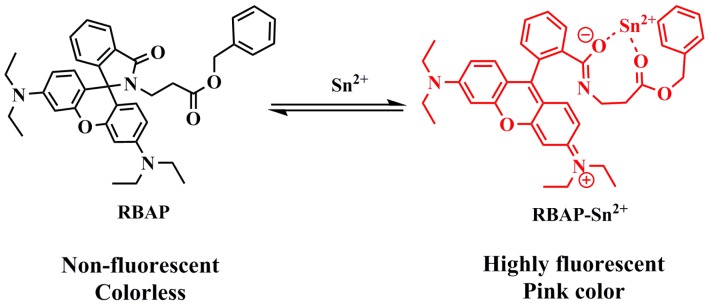
Proposed complexation mechanism of **RBAP** with Sn^2+^.

To further evaluate the selectivity of **RBAP** for Sn^2+^, the change in fluorescence intensity upon the addition of various metal ions under the same conditions was also investigated. The fluorescence spectra of **RBAP** (10 μM) in MeOH/H_2_O (2:3, v/v, pH 5.95) displayed a very weak fluorescence at 583 nm (λ_ex_ = 561 nm), indicating that the predominant form of **RBAP** was the spirolactam form. When Sn^2+^ (50 μM, 5 equiv.) was added to the **RBAP** solution, a fluorescence enhancement of greater than 300-fold was observed (Figure 8), indicating that the Sn^2+^ ion induced the formation of the ring-opened **RBAP**-Sn^2+^ complex, which exhibited strong fluorescence (Scheme 3) The solution changed from colorless to pink. (Figure 8 inset) other various metal ions (Mg^2+^, Sn^2+^, Cr^3+^, Ag^+^, Ca^2+^, Na^+^, Pb^2+^, K^+^, Mn^2+^, Zn^2+^, Cu^2+^, Cd^2+^, Li^+^, Ba^2+^, Fe^2+^, Co^2+^, Fe^3+^, Hg^2+^, Al^3+^ and Sn^4+^) did not induce any apparent fluorescence enhancement after the addition of five equiv. of the metal ions.

### 2.6. Fluorescence Titration of RBAP with Sn^2+^

**Figure 9:**
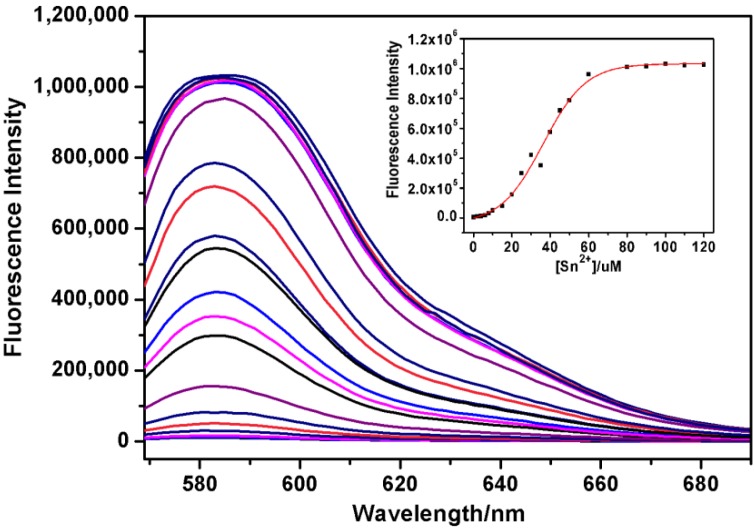
Changes in the fluorescence intensity of **RBAP** in MeOH/H_2_O (2:3, v/v, pH 5.95) upon the addition of Sn^2+^. λ_ex_ = 561 nm. **Inset**: Plot of fluorescence intensities at 583 nm upon the addition of Sn^2+^.

Figure 9 displays the fluorescence spectrum of **RBAP** in the presence of different concentrations of Sn^2+^. The free **RBAP** chemosensor (10 μM) exhibited a very weak fluorescence (λ_ex_ = 561 nm) at 583 nm. The titration of Sn^2+^ with **RBAP** led to a rapid increase in the emission intensity at 583 nm. Over a 300-fold fluorescence enhancement was observed under saturation conditions (10 equiv.). The association constant was also calculated using the following formula: F − F_0_ = △F = [Sn^2+^] (F_max_ − F_0_)/(1/K_a_ + [Sn^2+^]) based on a 1:1 stoichiometry, where F is the obtained fluorescence intensity, F_0_ is the fluorescence intensity of free **RBAP** at the emission wavelength, and F_max_ is the saturated fluorescence intensity for **RBAP**-Sn^2+^ complex. When the reciprocal of △F was plotted as a function of Sn^2+^ concentration, a linear relationship was obtained (y = A + Bx), and K_a_ was calculated from A/B. Therefore, the binding association constant for Sn^2+^, K_a_, was estimated to be 2.65 × 10^4^ M^−1^ in the MeOH/H_2_O (2:3, v/v, pH 5.95) solution, as inferred from the fluorescence titration curves of **RBAP** with the Sn^2+^ ion. The detection limit of Sn^2+^ by **RBAP** was determined to be 0.044 μM (Supporting Materials, S5).

### 2.7. NMR Study of RBAP with Sn^2+^

**Figure 10:**
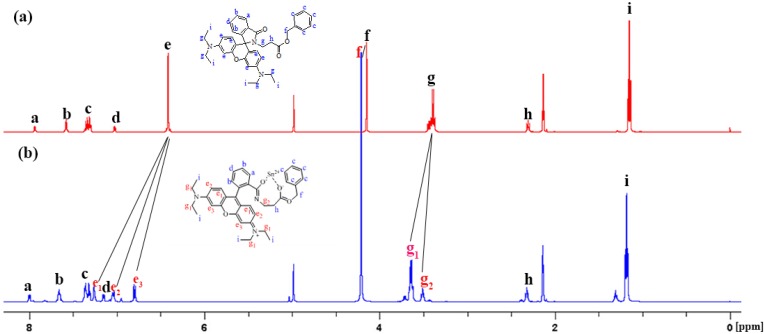
^1^H-NMR spectra (500 MHz, 298 K, acetone-*d*_6_:D_2_O (5:1, v/v) of (**a**) RBAP and (**b**) RBAP + 1 equiv. Sn^2+^.

To further elucidate the binding mode of **RBAP** with Sn^2+^, ^1^H-NMR spectra of **RBAP** in the presence and absence of Sn^2+^ were acquired. As shown in Figure 10, after the addition of 1 equiv. of Sn^2+^ into a solution of **RBAP** in acetone-*d_6_*:D_2_O (5:1, v/v), The singlet peak of H_f_ proton displayed a downfield shift from 4.15 ppm to 4.21 ppm (Δδ = 0.06 ppm), which originates from the coordination of the carbonyl oxygen of ester group on **RBAP** with Sn^2+^. In addition, the H_g_ multiplet, (the signals of H_g1_ and H_g2_ overlapped) displayed an apparent downfield shift and signal splitting from one multiplet peak centered at 3.41 ppm to two multiplet peaks centered at 3.65 ppm (Δδ = 0.24 ppm) and 3.50 ppm (Δδ = 0.09 ppm), and the singlet peak of the symmetric H_e_ proton also displayed a significant downfield shift and signal splitting, from a peak centered at 6.4 ppm to three doublets peaks centered at 6.78 ppm (Δδ = 0.38 ppm), 7.0 ppm (Δδ = 0.60 ppm) and 7.31 ppm (Δδ = 0.91 ppm), due to the Sn^2+^ ion-induced ring-opening process of the rhodamine B spirocycle. (Supporting Materials Figure S3). The above results indicate an interaction mode of **RBAP** and Sn^2+^ as proposed in Scheme 3, in which Sn^2+^ is coordinated with the two carbonyl oxygen atoms of **RBAP**.

### 2.8. Density Functional Theory (DFT) Calculations

**Figure 11:**
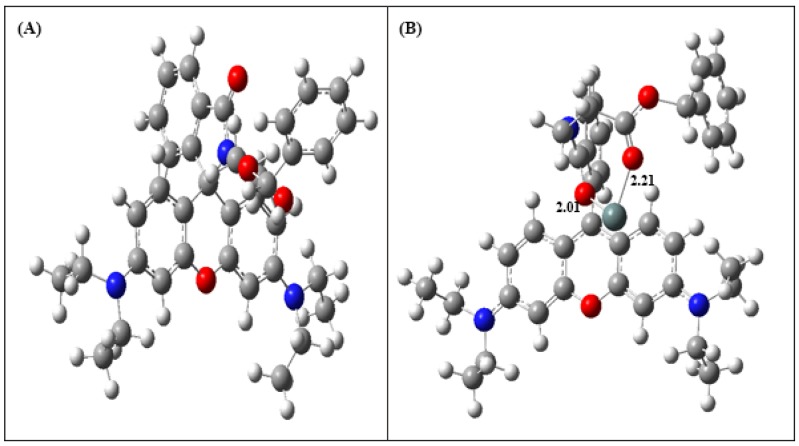
Energy-minimized structure of (**A**) **RBAP** and the (**B**) **RBAP**-Sn^2+^complex.

For better understanding of the nature of Sn^2+^ coordination with **RBAP**, energy optimized structures of **RBAP** and **RBAP**-Sn^2+^ (Figure 11) were obtained using DFT calculations with the B3LYP method using 6-31+G(d) as a basis set. 

**Figure 12:**
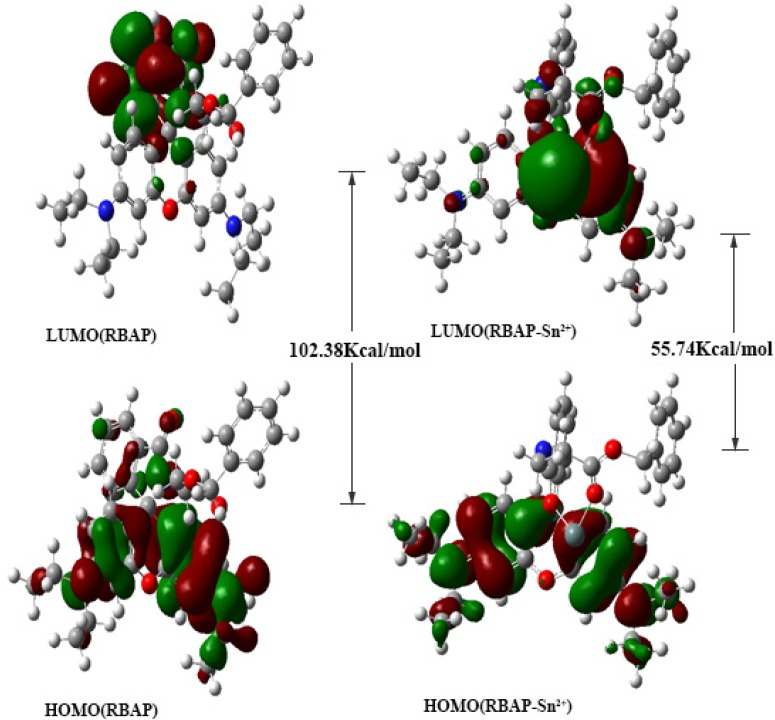
HOMO and LUMO orbitals of (**A**) **RBAP** and the (**B**) **RBAP**-Sn^2+^ complex.

The spatial distributions and orbital energies of the HOMO and LUMO of **RBAP** and **RBAP**-Sn^2+^ were also generated using DFT calculations (Figure 12). The results indicated that, in **RBAP** the HOMO is spread out on the xanthenes of the rhodamine B moiety, while the LUMO is centered on the spirocycle of the rhodamine B moiety and more localized. As a result, the C-N bond on the spirocycle of the rhodamine B moiety breaks to facilitate the binding of Sn^2+^ ions with the two **RBAP** carbonyl oxygen atoms. The π electrons in the HOMO of the **RBAP**-Sn^2+^ complex are mainly located in rhodamine B, while the LUMO is mostly located on the guest Sn^2+^ ion. The energy gap between the HOMO and LUMO was calculated to be 55.74 kcal/mol, an almost 50% decrease from that of **RBAP**, which was 102.38 kcal/mol (Supporting Materials). The results clearly suggest that the binding of Sn^2+^ to **RBAP** stabilizes the system because the calculated orbital energies of both the HOMO and LUMO were lowered for the complex. In the **RBAP**-Sn^2+^ complex, the distances of the two “Sn-O” bonds were calculated to be 2.01 and 2.21 Å, respectively (Figure 11).

## 3. Experimental Section

### 3.1. Materials and General Methods

All reagents and organic solvents were ACS grade or higher and were used without further purification. Unless otherwise noted, all chemicals were purchased from J&K Scientific (Shanghai, China) and were used as received. All solvents were of analytical grade, and double distilled water was used in all experiments. The salts used to prepare metal ion stock solutions were Ba(NO_3_)_2_, AgNO_3_, NaCl, LiCl·H_2_O, KCl, MgCl_2_·6H_2_O, FeCl_3_·6H_2_O, SnCl_2_·2H_2_O, CuCl_2_·2H_2_O, CaCl_2_, PbCl_2_, MnCl_2_·4H_2_O, ZnCl_2_, CdCl_2_·2.5H_2_O, FeCl_2_·4H_2_O, HgCl_2_, AlCl_3_, CoCl_2_·6H_2_O, SnCl_4_ and CrCl_3_·6H_2_O. The reactions were performed under an argon atmosphere using standard Schlenk techniques. Thin-layer chromatography was performed on a HAIYANG silica gel F254 plate, and compounds were visualized under UV light (λ = 254 nm). Column chromatography was performed using HAIYANG silica gel (type: 200–300 mesh ZCX-2).

^1^H (500 MHz) and ^13^C-NMR (126 MHz) spectra were recorded on an Avance 500 spectrometer (Bruker; Billerica, MA, USA). The chemical shifts are reported in δ units (ppm) downfield relative to the chemical shift of tetramethylsilane. The abbreviations br, s, d, t, and m denote broad, singlet, doublet, triplet, and multiplet, respectively. Mass spectra were obtained with a Finnigan TSQ Quantum LC/MS Spectrometer (Thermo Fisher Scientific Corp., Waltham, MA, USA). High-resolution mass spectra (HRMS) were acquired under electron ionization conditions with a double-focusing high-resolution instrument (Thermo Fisher Scientific Corp., Waltham, MA, USA) The pH levels of stock solutions were measured using a PHS-25C Precision pH/mV Meter (Aolilong, Hangzhou, China). UV-Vis and fluorescence spectra were obtained on a UV-3600 UV-VIS-NIR spectrophotometer (Shimadzu, Kyoto, Japan) and an Edinburgh FLS920 fluorescence spectrophotometer (Livingston, UK), respectively at room temperature.

### 3.2. Synthesis of 3-(Benzyloxy)-3-oxopropan-1-aminium chloride (**2**)

β-Alanine (2.9 g, 32.5 mmol) was suspended in 20 mL of phenylmethanol. Hydrogen chloride was passed through the solution for 15 min at room temperature. The reaction mixture was stirred at 120 °C for 12 h, cooled to room temperature, and then dried under vacuum. Flash chromatography (silica gel; MeOH/DCM, 5:95, v/v; R_f_ = 0.3) of the residue gave 2 as a white solid (6.45 g, 92%).^ 1^H-NMR (CDCl3): δ = 7.72 (s, 1H), 7.29–7.25 (m, 5H), 5.09 (s, 2H), 3.35 (t, *J* = 5.5 Hz, 2H), 2.92 (t, *J* = 6.5 Hz, 2H). 

### 3.3. Synthesis of Benzyl 3-(3',6'-bis(diethylamino)-3-oxospiro[isoindoline-1,9'-xanthen]-2-yl) propan oate (**RBAP**)

A solution of **2** (2.00 g, 10 mmol) and Et_3_N (1.5 mL, 10.8 mmol) in 20 mL CH_2_Cl_2_ was added to a solution of **Rhodamine B** (4.00 g, 8.3 mmol) and HOBt (1.2 g, 8.9 mmol) in 30 mL CH_2_Cl_2_. The reaction mixture was stirred at room temperature for 12 h, filtered through a pad of Celite and then dried under vacuum. Flash chromatography (silica gel; MeOH/DCM, 2/98, v/v; R_f_ = 0.3) of the residue gave **RBAP** as a bright-red solid (4.51 g, 90%). ^1^H-NMR (acetone-*d*_6_/D_2_O): δ = 7.94–7.92 (m, 1H), 7.58–7.56 (m, 2H), 7.36–7.29 (m, 5H), 7.03–7.01 (m, 1H), 6.43–6.39 (m, 6H), 4.98 (s, 2H), 3.45–3.37 (m, 10H), 2.30 (t, *J* = 7.75 Hz, 2H), 1.15 (t, *J* = 7.0 Hz, 12H) ppm. ^13^C-NMR (126 MHz, CDCl_3_, 298 K): δ = 171.6, 168.2, 153.9, 153.4, 148.9, 136.0, 132.5, 130.9, 128.9, 128.5, 128.2, 128.0, 123.8, 122.9, 108.3, 105.4, 98.2, 66.2, 65.0, 44.5, 35.9, 32.9, 29.8, 12.7 ppm. ESI-MS (m/s): Calculated for [M + H]^+^ C_38_H_42_N_3_O_4_^+^, 604.3170; HRMS found, 604.3180. (Supporting Materials, Figures S1–S3).

### 3.4. Experimental Procedure for X-ray Crystallographic Analysis

Crystallographic data of complexes were collected at 296 K on a Bruker APEX-II CCD system equipped with graphite-monochromated Mo-Kα radiation (λ = 0.071073 nm) using the ω-φ scan technique. Diffraction data were integrated using the SAINT program, which was also used for intensity corrections for Lorentz and polarization effects. A semi-empirical absorption correction was applied using SADABS. The structures were solved by direct methods, and all non-hydrogen atoms were refined anisotropically on F2 by full-matrix least-squares using the SHELXL-97 crystallographic software package. Summary of Data CCDC 969599, Compound Name: Formula: C_39_H_44_N_3_O_4_, Unit Cell Parameters: a 12.5467(15) b 22.338(3) c 12.0855(14) P21/c.

### 3.5. Stock Solution Preparation for Spectral Detection

Stock solutions (10^−2^ M) of the chlorides or nitrate salts of Mg^2+^, Sn^2+^, Cr^3+^, Ag^+^, Ca^2+^, Na^+^, Pb^2+^, K^+^, Mn^2+^, Zn^2+^, Cu^2+^, Cd^2+^, Li^+^, Hg^2+^, Al^3+^, Ba^2+^, Fe^2+^, Co^2+^, Sn^4+^ and Fe^3+^ in MeOH/H_2_O (2:3, v/v) were prepared. Stock solutions (10^−3^ M) of **RBAP** were prepared in THF/methanol/H_2_O (5:2:3, v/v/v). **RBAP** working solutions were freshly prepared by diluting the high concentration stock solution to the desired concentration prior to spectroscopic measurements.

### 3.6. Computational Studies

Computational studies were carried out to investigate the nature of Sn^2+^ coordination with **RBAP** using the Gaussian software package. All geometries for **RBAP** and **RBAP**-Sn^2+^ were optimized using *ab initio* HF and density functional theory (DFT) calculations. The geometries were first optimized at the HF/3-21G level. The resulting structures were further optimized by DFT calculations using B3LYP with larger basis sets, *i.e.*, the SDDAll basis set for Sn^2+^ and 6-31+G(d) for all other atoms in the complex.

## 4. Conclusions

In conclusion, we report a rhodamine B derivative, **RBAP**, which is a selective and sensitive chemosensor that specifically recognizes the Sn^2+^ ion in a MeOH/H_2_O (2:3, v/v, pH 5.95) solution by UV/vis and fluorescence spectroscopy. The chemical structure of **RBAP** was analyzed by ^1^H-NMR, ^13^C-NMR, and HRMS, and its structure was further confirmed by X-ray analysis. The 1:1 coordination mode was proposed on the basis of Job’s plot. The Sn^2+^ binding ability of **RBAP** was further demonstrated by DFT calculations, which suggested that both the HOMO and LUMO orbitals in the **RBAP**-Sn^2+^ complex were stabilized and that the optical detection resulted from the significant decrease in the HOMO-LUMO energy gap.

## Supplementary Materials

Supplementary materials can be accessed at: http://www.mdpi.com/1420-3049/19/6/7817/s1.
