# Supplementary material for: RBAP, a Rhodamine B-Based Derivative: Synthesis, Crystal Structure Analysis, Molecular Simulation, and Its Application as a Selective Fluorescent Chemical Sensor for Sn2+
Source: Molecules. 2014 Jun 11;19(6):7817–31. doi: 10.3390/molecules19067817 (PMC6271026; doi:10.3390/molecules19067817)
Supplement: Supplementary file 1 [file molecules-19-07817-s001.pdf]

Supplementary Materials

^{1}H-NMR of **RBAP** in acetone-d_6:D_2O (5:1, v/v) S2
^{13}C-NMR of **RBAP** in $CDCl_3$ S3
HRMS of **RBAP** S4
Crystallographic data of **RBAP** S5
Association constant calculations S5
Determination of detection limit S6
Absorbance of **RBAP** at 561 nm as a function of Sn^{2+} concentration S7
Fluorescence intensities of **RBAP** at 583 nm upon the addition of Sn^{2+} S7
HOMO-LUMO energy calculations for **RBAP** and the **RBAP**-Sn^{2+} complex S8

Figure S1. ^{1}H-NMR of **RBAP** in acetone-d_6:D_2O (5:1, v/v).

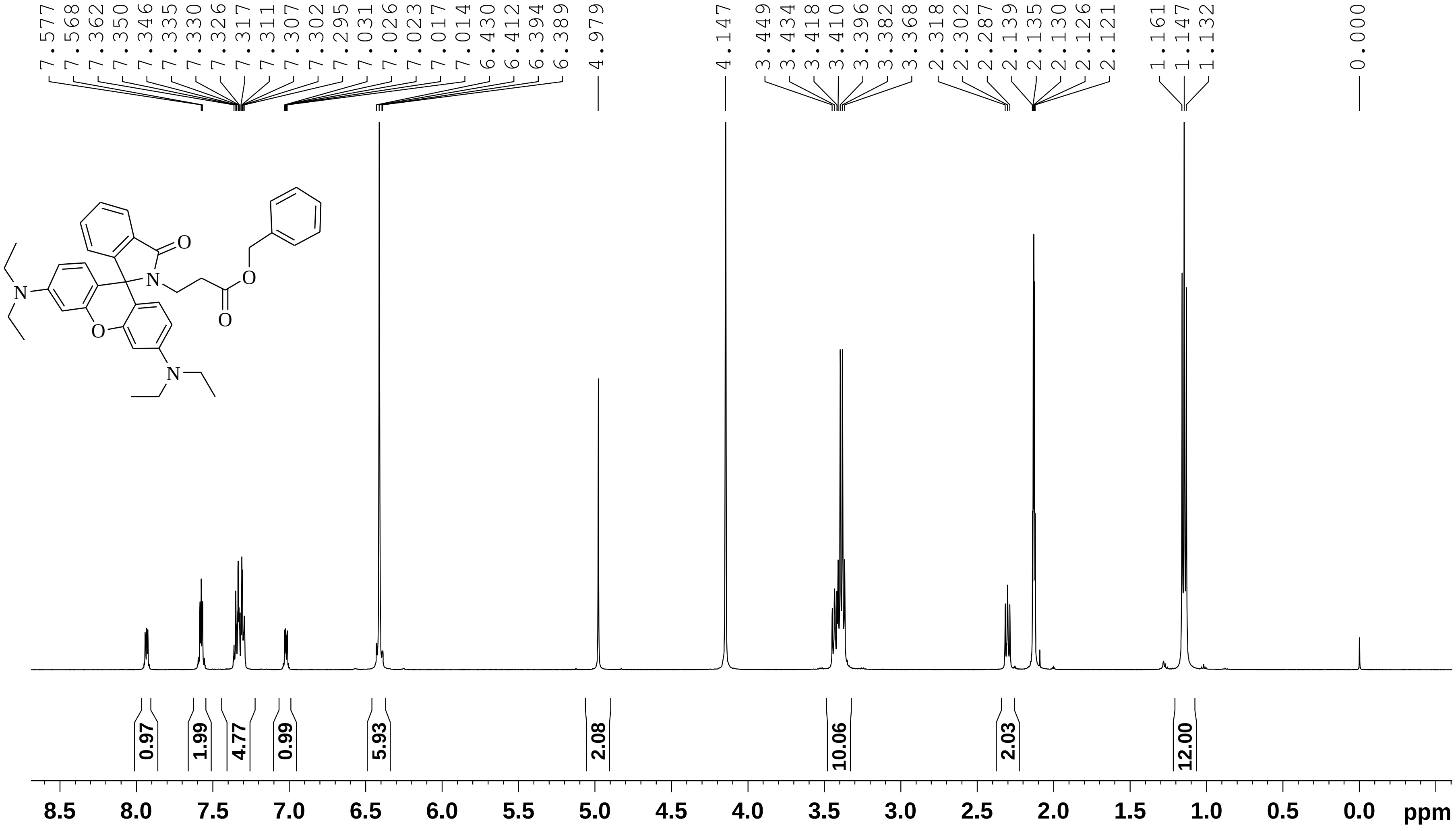

Figure S2. ^{13}C-NMR of **RBAP** in $CDCl_3$.

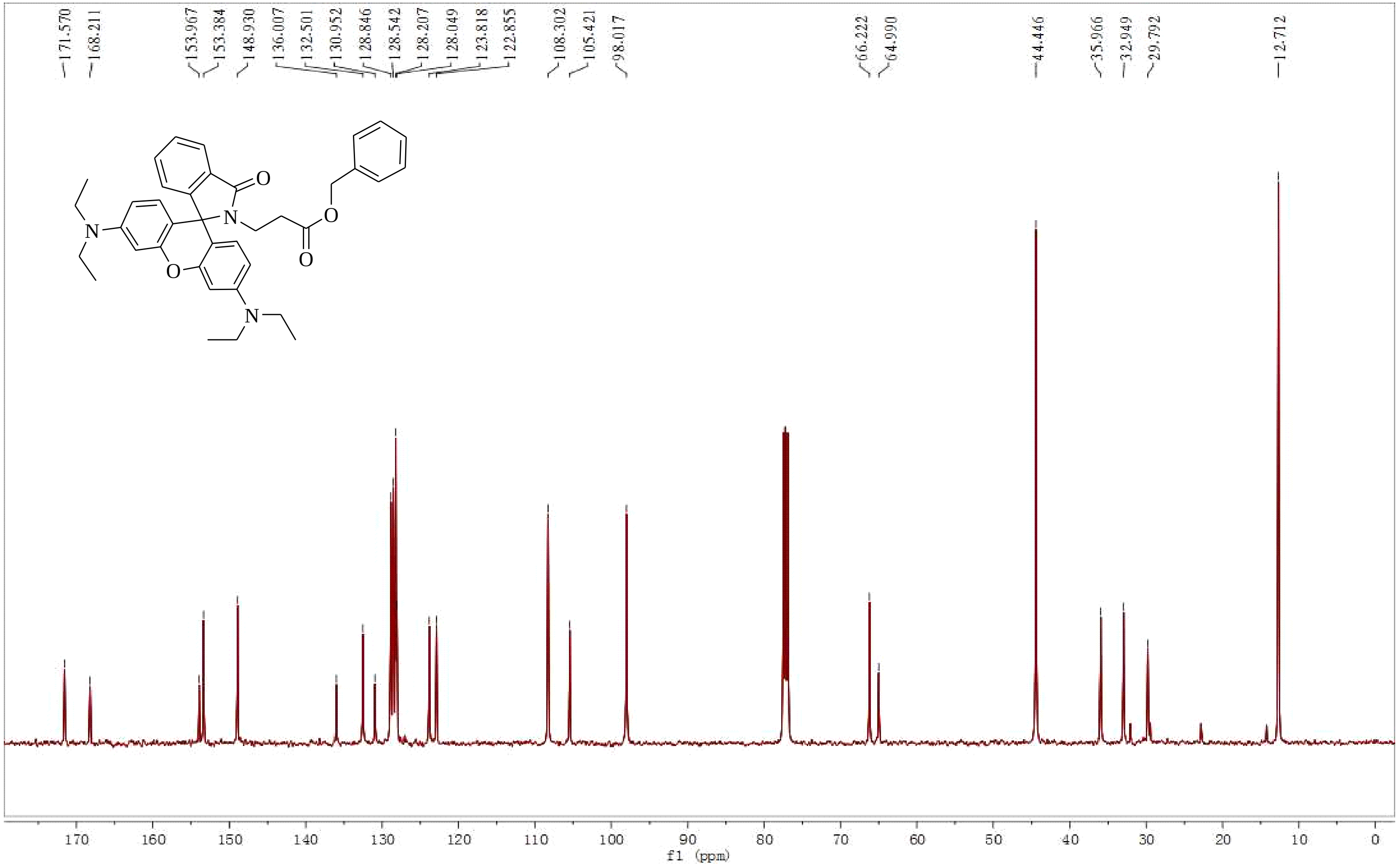

Figure S3. HRMS of RBAP.

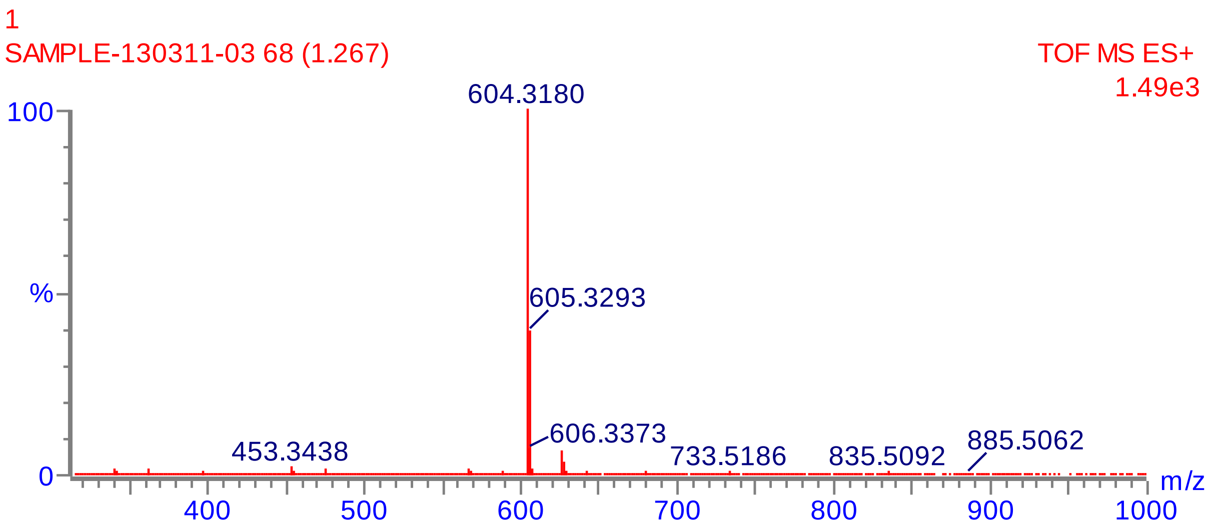

Crystallographic data of RBAP

[a] $w = 1/[\sigma^2(F_0)^2 + (0.0893P)^2 + 1.6528P]$, where $P = (F_0^2 + 2F_c^2)/3$

complex	**RBAP**
Formula	$C_{38}H_{41}N_3O_4$
Formula weight	603.74
Crystal system	Monoclinic
space group	*P*2(1)/C
a (Å)	12.5467(15)
b (Å)	22.338(3)
c (Å)	12.0855(14)
α (°)	90.00
β (°)	103.278(2)
γ (°)	90.00
Volume(Å^3)	3296.6(7)
Z	4
T, (K)	296(2)
μ (mm^{-1})	0.079
D_{calcd} (g/m^3)	1.216
F(000)	1288
Reflections collected	6128
Unique reflections	4350
Goof	1.036
R_1[I> 2σ(I)]	0.0645
wR_2[I>2σ(I)]	0.1745[a]
CCDC	CCDC 969599

Calculation of Association Constant

The apparent association constant was calculated by the following formula: $F - F_0 = \Delta F = [Sn^{2+}](F_{max} - F_0)/(1/Ka + [Sn^{2+}])$, where F is the obtained fluorescence intensity of **RBAP** with different equivalent of Sn^{2+} at the emission wavelength, F_{max} is the saturated fluorescence intensity of **RBAP** with different equivalent of Sn^{2+} at the emission wavelength, and F_0 is the fluorescence intensity of **RBAP** at the emission wavelength. Plot $1/\Delta F$ against $1/[Sn^{2+}]$ to a linear relation formula (y = A + Bx, Figure S4). Ka was calculated from $A/B = 7.96893 * 10^{-6}/3.00674 * 10^{-10} = 2.65 * 10^4$ M^{-1}. (Table S1, Figure S4).

Table S1. Detailed Calculations for Ka.

$[Sn^{2+}]$(M)	$1/[Sn^{2+}]$	F	F − F0	1/(F − F0)
0		5000(F_0)		
1.00×10^{-5}	1.00×10^{5}	5.11×10^{4}	4.61×10^{4}	2.17×10^{-5}
1.50×10^{-5}	66,666.67	8.06×10^{4}	7.56×10^{4}	1.32254×10^{-5}
$2.00 \times 10^{-5}5$	50,000	1.56×10^{5}	1.51×10^{5}	6.62291×10^{-6}
2.50×10^{-5}	40,000	2.99×10^{5}	2.94×10^{5}	3.4007×10^{-6}
3.00×10^{-5}	33,333.33	4.21×10^{5}	4.16×10^{5}	2.40186×10^{-6}

Figure S4. Plot 1/△F against $1/[Sn^{2+}]$ to a linear relation formula (y = A + Bx).

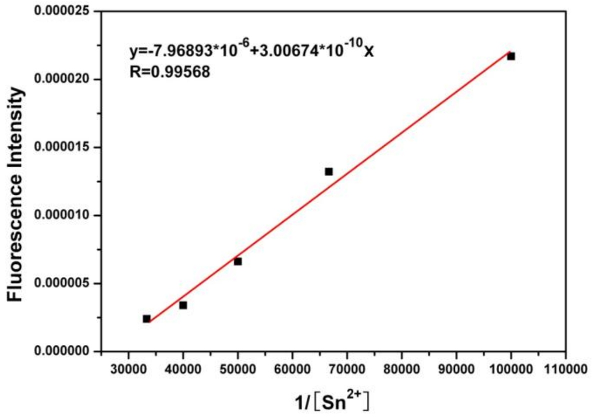

Determination of Detection Limit

The detection limit of RBAP for Sn^{2+} was determined from the following equation: DL = K * (SD/S), where K = 3; SD is the standard deviation of the blank solution detected for 5 times; S is the slope of the calibration curve. SD value calculated from standard deviation of the blank solution (RBAP (10 μM) in a methanol/H2O (2:3, v:v)) is 66.14 (Table S3). From Figure S5 we get slope = 4515. Thus, using the formula we get the Detection Limit = 3 × (66.14/4515) = 0.044 μM.

Figure S5. Fluorescence intensity at 583 nm of RBAP (10 μM) as a function Sn^{2+} concentration.

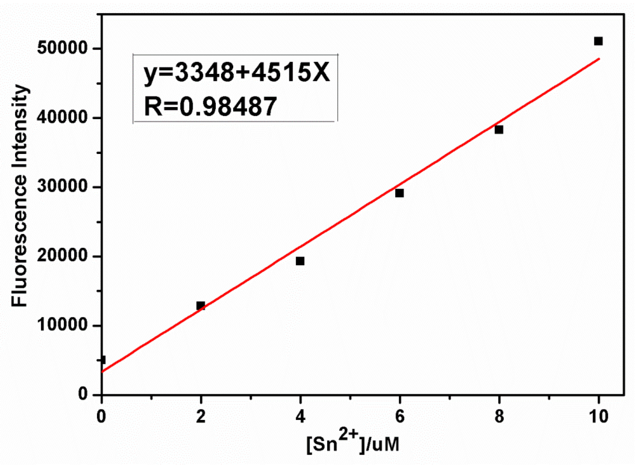

Table S2. Calculation for $\mathbf{SD} = \sqrt{\frac{1}{N-1}\sum_{i=1}^{N}(X_i - \bar{X})^2}$.

F.I. of the Blank Solution		$X_i - \bar{X}$ (I = 1, 2, 3, 4, 5)	$(X_i - \bar{X})^2$		SD
X_1	5124	76.4	Y_1	5836.96	
X_2	4963	−84.6	Y_2	7157.16	
X_3	5057	9.4	Y_3	88.36	
X_4	5000	−47.6	Y_4	2265.76	
X_5	5094	46.4	Y_5	2152.96	
**average value** $\bar{X}$	5047.6		$SD^2 = (Y_1 + Y_2 + Y_3 + Y_4 + Y_5)/4$	4375.25	66.14

Figure S6. Absorbance of **RBAP** at 561 nm as a function of Sn^{2+} concentration.

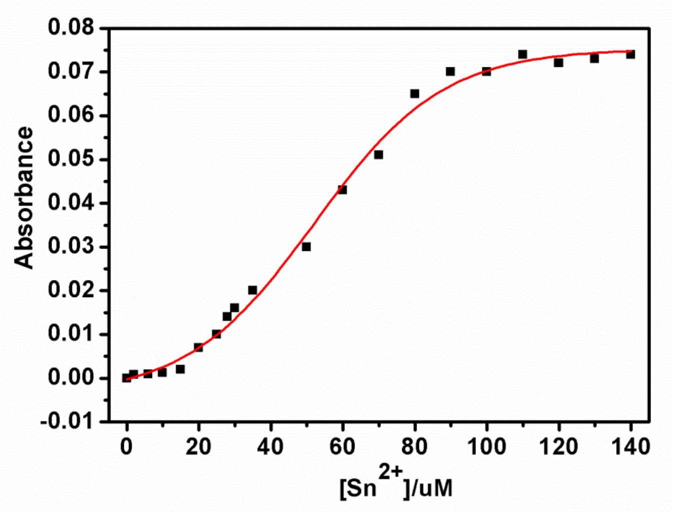

Figure S7. Fluorescence intensities of **RBAP** at 583 nm upon the addition of Sn^{2+}.

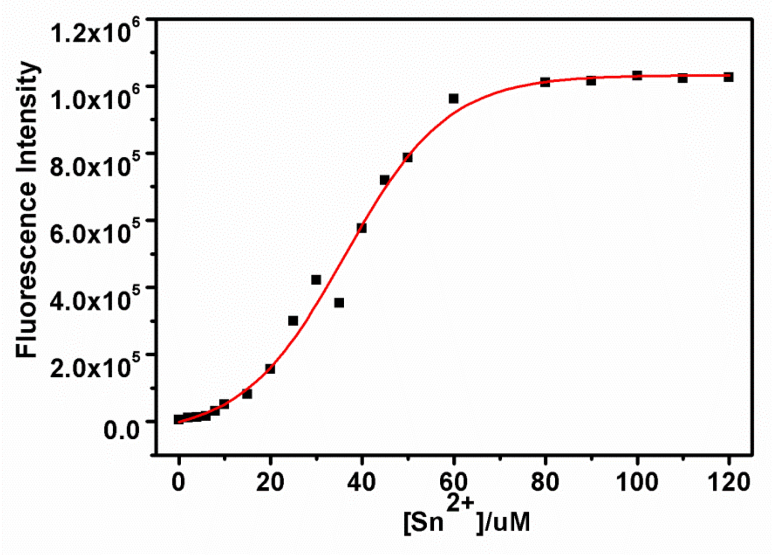

Table S3. HOMO-LUMO energy calculated for **RBAP** and the **RBAP**-Sn^{2+} complex.

**Species**	**E(HOMO)**	**E(LUMO)**	**ΔE (Hartree)**	**ΔE (kcal/mol)**
**RBAP**	−0.19	−0.03	0.16	102.38
**RBAP**-Sn^{2+}	−0.38	−0.30	0.09	55.74

© 2014 by the authors; licensee MDPI, Basel, Switzerland. This article is an open access article distributed under the terms and conditions of the Creative Commons Attribution license (http://creativecommons.org/licenses/by/3.0/).
